# Prescriptions

**Published:** 1896-12

**Authors:** 


CHORDEE.

℞ Ext. opii aquos............gr. ij
M. Pulv. camphoræ .........gr. iv
In pil. no. xii div.........
Sig: One or both on retiring.—*Van Buren and Keyes.*

PNEUMONIA.

℞ Tinct. aconiti rad...........ʒij
M. Tinct. opii.................ʒiij
Sig: Thirteen drops at once, followed by five drops every hour or two. (*In the stage of congestion.*)—*Bartholow.*

RHEUMATISM, CHRONIC.

℞ Potassii et sokii tartratis... ℥ss

Vini colchici sem...........ʒij

M. Aquæ...............q. s. ad ℥ij

Sig: A teaspoonful thrice daily.—*Charity Hospital, N. Y.*

HERPES ZOSTER.

℞ Boric acid...............gr. 1
Glycerine, q. s ...........
Vaseline....................gr. 30
M. Cocaine hydrochlorate, Extract of opium, } aa...ctgr. 30
Sig: Apply to the affected part. The neuralgia following the eruption is best treated by Fowler's solution.

BRONCHITIC ASTHMA.

℞ Potassii iodidi..............ʒij

Ammon, carb...............ʒj

Tinct. lobeliæ............ .fʒij

Sp. chloroformi............fʒiv

Vin. ipecac................fʒj

M. Infus. senegæ, q. s. ad.....f℥vj

Sig: A tablespoonful in a wineglassful of water every four hours.

